# Healthcare workers and prevention of hepatitis C virus transmission: exploring knowledge, attitudes and evidence-based practices in hemodialysis units in Italy

**DOI:** 10.1186/1471-2334-13-76

**Published:** 2013-02-07

**Authors:** Aida Bianco, Francesca Bova, Carmelo GA Nobile, Claudia Pileggi, Maria Pavia

**Affiliations:** 1 Department of Health Sciences, Medical School, University of Catanzaro “Magna Græcia”, Via T. Campanella, 115, Catanzaro, 88100, Italy

**Keywords:** Evidence-based practices, Hemodialysis, Hepatitis C virus, Italy, Nurses, Prevention

## Abstract

**Background:**

Evidence exists regarding the full prevention of HCV transmission to hemodialysis patients by implementing universal precaution. However, little information is available regarding the frequency with which hospitals have adopted evidence-based practices for preventing HCV infection among hemodialysis patients. A cross-sectional survey has been conducted among nurses in Calabria region (Italy) in order to acquire information about the level of knowledge, the attitudes and the frequencies of evidence-based practices that prevent hospital transmission of HCV.

**Methods:**

All 37 hemodialysis units (HDU) of Calabria were included in the study and all nurses were invited to participate in the study and to fill in a self-administered questionnaire.

**Results:**

90% of the nurses working in HDU participated in the study. Correct answers about HCV pattern of transmission ranged from 73.7% to 99.3% and were significantly higher in respondents who knew that isolation of HCV-infected patients is not recommended and among those who knew that previous bloodstream infections should be included in medical record and among nurses with fewer years of practice. Most correctly thought that evidence-based infection control measures provide adequate protection against transmission of bloodborne pathogens among healthcare workers. Positive attitude was significantly higher among more knowledgeable nurses. Self-reporting of appropriate handwashing procedures were significantly more likely in nurses who were aware that transmission of bloodborne pathogens among healthcare workers may be prevented through adoption of evidence-based practices and with a correct knowledge about HCV transmission patterns.

**Conclusions:**

Behavior changes should be aimed at abandoning outdated practices and adopting and maintaining evidence-based practices. Initiatives focused at enabling and reinforcing adherence to effective prevention practices among nurses in HDU are strongly needed.

## Background

Chronic hepatitis C virus (HCV) infection is a major cause of liver disease and hepatocellular carcinoma all over the world, it accounts for more than 350 000 deaths each year, and it is estimated that 130–170 million people are chronically infected with HCV [1]. Even if the number of new cases of acute HCV infection is declining, as a result of the identification, in 1989, of HCV and the advent of serological screening of blood and blood products, HCV continues to be a major disease burden in the world [2].

Although the epidemiology of HCV infection in patients receiving maintenance dialysis has considerably changed, there is still a particular concern because of the high risk for chronic liver disease, complications in renal transplantation, and death [3,4]. The main mechanism of nosocomial infection by HCV in hemodialysis (HD) patients is suboptimal adherence to infection-control recommendations against bloodborne pathogen agents [5]. Previous studies demonstrated that healthcare workers (HCW) inconsistencies in attitudes and practices about bloodborne viruses exist [6-8], and a recent study conducted in chronic hemodialysis units (HDU) in the United States has provided epidemiologic evidence of associations between specific patient-care practices and higher HCV infection prevalence among HD patients [9].

Various evidence-based guidances have been published for the prevention of healthcare-associated infections (HAI) in all HD settings in order to globalize and share the evidence [10-13]. However, little information is available regarding the frequency with which hospitals have adopted evidence-based practices for preventing HCV infection among HD patients. A survey of 300 HDU in Japan, revealed that policy regarding infection control and prevention varies considerably among Japanese HDU and more than 50% reported that they did not comply with recommendations about places for preparing medications and clean areas in the units [14]. Analogous results were shown by a cross-sectional survey carried out among 420 US HDU staff members who displayed low compliance with recommended hand hygiene and glove use practice [15]. On the other hand, evidence exists regarding the full prevention of HCV transmission to HD patients by implementing universal precautions [16].

Therefore, a cross-sectional survey has been conducted among nurses who are in practice in chronic HDU in Calabria region (Italy) in order to acquire and make available information about the level of knowledge, the attitudes and the frequencies of evidence-based practices and those determinants that may help expand understanding on how to motivate HCWs to adopt precautions to prevent HDU transmission of HCV.

## Methods

The cross-sectional survey was conducted from December 2011 to March 2012.

Prior to commencement of the study, head physician of all 37 HDU in Calabria region (Italy) were contacted to present the project and to ascertain the exact number of working nurses. Then, all nurses working in the HDU were invited to participate in the study. A sufficient number of self-administered questionnaires was mailed to the HDU, with a letter containing information about the purpose of the survey.

The participation in the survey was completely voluntary and anonymous, and it was assured that data collected would be used solely for fulfilling the research aim. Second and third questionnaires for non responders were mailed. In an attempt to maximize the response rate, follow-up letters and telephone calls to encourage participation were made periodically to all HDU until February 2012.

No financial or material incentives for participation in this study were used.

Two physicians trained in infection control tested the face validity and comprehensibility of the instrument. All judges had to be in full agreement for any item to be included, and their comments were taken into account in constructing the final questionnaire. A pilot study was conducted for evaluating the data-collection process and usefulness of the questionnaire. After the pilot study, the tool was revised.

Two questionnaires were designed: one addressed to the head nurse and one to all other nurses. Both questionnaires included the following 5 main sections focusing on a) nurses’ demographic and practice characteristics; b) infection-control issues (programs in preventing HAI, whether they refer to published guidelines on the prevention of HAI); c) knowledge about HCV infection (transmission patterns, prevention strategies, isolation of HCV-infected patients, use of dedicated dialysis machines, routine serologic testing); d) attitudes about risk of contracting HCV infection in HDU, adherence to universal precaution, handwashing and use of gloves; e) practices about handwashing, use and replacement of gloves, disinfection procedures, handling of nondisposable items and monitoring equipment, medications preparation, use of personal protective equipment. In addition, the questionnaire addressed to the head nurses comprised a sixth section, that included a set of nine questions focusing on the characteristics of the HDU (Additional file 1: Questionnaire).

Correct answers to each item were based on a review of the available literature as well as policies and guidelines [9-13].

Each section elicited responses in a variety of formats: closed-ended questions with multiple possible answers, the 5-point Likert scale and open option questions.

Reliability of the tool was checked through an additional administration for 40 nurses at a time interval of 20 days by the first administration and tested using the weighted-kappa which showed satisfactory test-retest reliability for the questionnaire score (kappa scores >0.7).

The study protocol was approved by the Ethics Committee of the “Mater Domini” Hospital of Catanzaro (Italy) (15/12/2011).

### Statistical analysis

Models were constructed in order to test the following hypotheses: 1) several practice characteristics, such as years in practice and specific education programs provided at the dialysis facilities might be determinants of knowledge and that several aspects of knowledge might be associated to each other; 2) solid knowledge and some practice characteristics might be determinants of positive attitudes; 3) knowledge and positive attitudes might be determinants of correct practices towards prevention of HCV. Bivariate analysis and multivariable stepwise logistic regression models were constructed in order to determine significant independent predictors of the following outcomes of interest: knowledge about HCV transmission patterns (having correctly identified all HCV modes of transmission vs all others) (Model 1); positive attitude toward handwashing and wearing gloves (belief that wearing gloves does not replace the need of handwashing vs all others) (Model 2); appropriate handwashing procedure (washing hands immediately before gloves are worn and after gloves are removed vs all others) (Model 3). The outcome variables originally consisting of multiple categories were collapsed into two levels. In all models, the following explanatory variables were included: years in practice (ordinal, years: ≤ 14=0, 15-23=1, ≥ 24=2), having received information about strategies to prevent HAI at the time of employment (no=0, yes=1), correct knowledge about HCV prevention (avoid sharing razors or toothbrushes and use of condom when having sex) (no=0, yes=1), knowledge about isolation of HCV-infected patients (no=0, yes=1). Satisfaction with medical equipment available in the HDU (clamps, blood pressure cuffs, trays, tourniquets, etc.) (dissatisfied=0, satisfied=1) and knowledge that previous bloodstream infections should be included in medical record (no=0, yes=1), were also included in Model 1; knowledge about HCV transmission patterns (no=0, yes=1) in the Model 2 and 3. Awareness that transmission of bloodborne pathogens among HCWs may be prevented through adoption of evidence-based practices (disagree=0, agree=1), belief that wearing gloves does not replace the need of handwashing (disagree=0, agree=1), and awareness that handwashing between patient contacts reduces the risk of HCV infection (disagree=0, agree=1), were also included in Model 3.

The results of the logistic regression analysis are presented as odds ratios (ORs) and 95% confidence intervals (CIs). A two-sided *p*-value for all tests of 0.05 and less was considered as indicating a statistically significant difference. We constructed a backward stepwise multivariate logistic regression model and the significant level for variables entry the model was set at 0.2 and for removal at 0.4.

Stata version 11 statistical software package was used in conducting all data analysis [17].

## Results

All 37 HDU in the region agreed to participate in the study. All HDU were no profit, since in Italy universal and free access to primary health care is provided through the National Health Service and most (62.2%) were hospital affiliated. The mean number of HDU beds was 11.7 (range 4–32) and 76.5% reported two scheduled shifts of dialysis per day and the mean number of patients per shift was 11.4 (± 5.6 SD). In 63% of HDU, samples of blood and other biological fluids are collected in a separate area and sufficient supplies of gloves (100%), protective glasses (86.7%), masks (83.3%) and aprons (66.7%) were available. An HAI prevention written policy and continuing education programs about strategies to prevent them are available in 52.2% and 37.9% of HDU, respectively.

Of the original sample of 373 nurses, 11 were ineligible because they were not in clinical practice. For the remaining 362 nurses, a total of 326 returned the questionnaire for a response rate of 90%. The mean age of participants was 45.6 (± 8.4 SD) years, 54% were females, the mean number of years in practice in hospital and in HDU was 19.1 (± 9.5 SD) and 11.7 (± 9.2 SD), respectively, and 65.7% of the sample was satisfied about medical equipment provided.

Table 1 summarizes the data concerning the level of knowledge about HCV infection. Only 49.8% correctly identified all 8 investigated modes of transmission. Almost all (93.9%) identified receiving a blood transfusion from an infected donor, (91.4%) having sex with a HCV-positive partner and (90.7%) sharing needles while injecting drugs, as a certain transmission route. Surprisingly, respondents overestimated the risk of infection by HCV, since 11.5% believed that it can be transmitted by kissing an HCV positive subject. Moreover, 19.2% of respondents did not indicate getting a tattoo as a mode of transmission of HCV. A large proportion of respondents (98.7%) knew that avoiding sharing razors or toothbrushes is a preventive measure for HCV transmission but only 63.4% of the sample also knew the other preventive measure investigated (condom use when having sex), and 21.4% wrongly believed avoiding breastfeeding is a practice to reduce the risk of HCV transmission.

**Table 1:** Knowledge about HCV infection in HDU

	**Correct answer**	
	**N**	**%**
**Knowledge**		
*Transmission patterns*		
Hugging an HCV-positive individual (313)	311	99.4
Receiving a blood transfusion from an infected donor (313)	294	93.9
Ingestion of HCV-contaminated food (313)	291	93.0
Having sex with an HCV-positive partner (313)	286	91.4
Sharing needles while injecting drugs (313)	284	90.7
Kissing an HCV-positive individual (313)	277	88.5
Getting a tattoo (313)	253	80.8
Being born to an HCV-positive mother (313)	230	73.5
*HCV prevention*		
Avoid sharing razors or toothbrushes (309)	305	98.7
Avoid pregnancy (309)	290	93.9
Avoid kidney transplant (309)	288	93.2
Use a condom when having sex (309)	284	91.9
Avoid breastfeeding (309)	243	78.6
*HCV seropositivity*		
Include routine serologic testing for HBV and HCV infections in medical record (323)	318	98.4
Perform testing for HCV when patient first start hemodialysis (317)	311	98.1
Perform testing for HCV for hemodialysis patients periodically (311)	305	98.1
Include vaccination against HBV in medical record (316)	309	97.8
Include previous bloodstream infections in medical record (280)	228	81.4
Do not isolate HCV-infected patients (316)	96	30.4
Use dedicated dialysis machines for HCV infected patients (321)	30	9.4

Nurses were asked about the importance of a certain aspect of HCV seropositivity and almost all (98.1%) agreed that testing for HCV when patient first start HD is an effective infection-control procedure to prevent transmission of bloodborne pathogens. Only 30.4% reported that isolation of HCV-infected patients is not recommended as an alternative to strict infection-control procedures. Table 2 presents the distribution of correct knowledge about HCV transmission patterns according to various explanatory variables. The correct knowledge is associated with the nurse’s practice characteristics, since it significantly decreased with the increasing number of years in practice (χ^2^=8.26, 2 df, p=0.016), and with correct knowledge about isolation of HCV-infected patients (χ^2^=5.72, 1 df, p=0.017). The results of the multivariate logistic regression analysis showed that knowledge about HCV transmission patterns was significantly higher among respondents who knew that isolation of HCV-infected patients is not recommended (OR=2.72; 95% CI 1.55-4.78) and among those who knew that previous bloodstream infections should be included in medical record (OR=2.68; 95% CI 1.3-5.54). Moreover, knowledge was greater among nurses with a lower number of years in practice (OR=0.68; 95% CI 0.49-0.94) (Model 1 in Table 2).

**Table 2:** Bivariate and multivariate analyses results

	**Correct knowledge about HCV trasmission patterns**				**Positive attitude toward handwashing and wearing gloves**				**Appropriate handwashing procedures**			
	**Bivariate**		**Multivariate**		**Bivariate**		**Multivariate**		**Bivariate**		**Multivariate**	
			**Log-likelihood=−154.88**				**Log-likelihood=−61.68**				**Log-likelihood=−154.55**	
			**χ2= 23.84 (4 df), p<0.001**				**χ2= 25.49 (4 df), p<0.001**				**χ2= 30.52 (4 df), p<0.001**	
**Variable**	N	%	OR	95% CI	N	%	OR	95% CI	N	%	OR	95% CI
**Years in practice**												
≤14	59	60.8			91	91.9			68	68		
15-23	53	51	0.68^#^	0.49-0.94	96	89.7	0.73^#^	0.41-1.3	62	59.6	†	†
*≥*24	44	40.7			101	91.8			67	59.8		
	χ^2^ for trend=8.23, *p*=0.004				χ^2^ for trend=0.40, *p=*0.995				χ^2^ for trend=1.99, *p=*0.229			
**Information about strategies to prevent healthcare associated infections at the time of employment**												
No	39	42.4			86	87.8			56	57.7		
Yes	109	54	†	†	185	92.5	†	†	135	67.5	†	†
	χ^2^ = 3.38, 1 df, *p=*0.066				χ^2^ = 1.80, 1 df, *p=*0.180				χ^2^ = 2.72, 1 df, *p=*0.099			
**Knowledge about HCV prevention**												
Incorrect	63	55.8			97	86.6	1.00^§^		70	62.5		
Correct	91	46.4	†	†	185	95.8	5.8	2.06-16.29	125	64.8	†	†
	χ^2^ = 2.49, 1 df, *p=*0.114				χ^2^ = 8.69, 1 df, *p=*0.003				χ^2^ = 0.16, 1 df, *p=*0.691			
**Knowledge about isolation of HCV-infected patients**												
Incorrect	75	43.4	1.00^§^		158	87.3	1.00^§^		104	57.8	1.00^§^	
Correct	76	57.1	2.72	1.55-4.78	126	96.9	4.04	1.12-14.6	93	71.5	1.52	0.85-2.71
	χ^2^ = 5.72, 1 df, *p=*0.017				Fisher’s exact *p=*0.004				χ^2^ = 6.17, 1 df, *p=*0.013			
**Knowledge that previous bloodstream infections should be included in medical record**												
Agree	120	54.1	2.68	1.3-5.54	199	89.2	*	*	151	66.8	*	*
Disagree	20	39.2	1.00^§^		49	96.1			29	58		
	χ^2^ = 3.65, 1 df, *p=*0.056				Fisher’s exact *p=*0.186				χ^2^ = 1.4, 1 df, *p=*0.236			
**Satisfaction about medical equipment**												
Dissatisfied	59	56.7	1.00^§^		-	-			-	-		
**S**atisfied	95	47.7	0.65	0.37-1.15			*	*			*	*
	χ^2^ = 2.21, 1 df, *p=*0.137											
**Knowledge about HCV transmission patterns**												
Incorrect												
Correct	-	-			138	88.5	1.00^§^		84	54.2	1.00^§^	
			*	*	147	96.1	3.65	1.23-10.81	112	73.2	2.17	1.25-3.77
					Fisher’s exact *p=*0.018				χ^2^ = 12.02, 1 df, *p=*0.001			
**Awareness that transmission of bloodborne pathogens among healthcare workers may be prevented through adoption of evidence-based practices**												
Agree	-	-	*	*	-	-	*	*	169	69.5	3.02	1.6-5.69
Disagree									26	43.3	1.00^§^	
									χ^2^ = 14.41, 1 df, *p*<0.001			
**Belief that wearing gloves does not replace the need of handwashing**												
Agree	-	-	*	*	-	-	*	*	13	46.4	1.00^§^	
Disagree									185	64.5	1.96	0.76-5.09
									χ^2^ = 3.55, 1 df, *p=*0.059			
**Awareness that handwashing between patients contacts reduces the risk of HCV infection**												
Agree	-	-	*	*	-	-	*	*	163	63.2	1.52	0.76-3.01
Disagree									34	60.7	1.00^§^	
									χ^2^ = 0.11, 1 df, *p=*0.730			

- not tested; * not included in the model; † removed by the model;^#^ordinal variable; ^§^ reference category.

Table 3 reports the results on nurses’ attitudes towards several aspects of HCV infection prevention. Nurses’s fear of contagion by a patient with HCV was indicated by a vast majority (97.8%) of respondents who believed that they are at risk of becoming infected with HCV by working in HDU. By contrast, only 70.8% of respondents agreed that HCV can be spread from patient-to-patient in the HDU. Most (80%) correctly thought that evidence-based infection-control measures provide adequate protection against transmission of bloodborne pathogens among HCWs. The majority (91.3%) of the sample agreed that wearing gloves does not replace the need of handwashing, and a significant association with positive attitude toward handwashing and wearing gloves was found, as expected, for correct knowledge about HCV prevention (χ^2^=8.69, 1 df, p=0.003), isolation of HCV-infected patients (Fisher’s exact p=0.004), and HCV transmission patterns (Fisher’s exact p=0.018) (Table 2). The results of the multiple logistic regression analysis, with the belief that wearing gloves does not replace the need of handwashing as the dependent variable, substantially confirmed the findings of the bivariate analysis: the nurses with that positive attitude were more likely to know how to prevent HCV infection (OR=5.8; 95% CI 2.06-16.29), that isolation of HCV-infected patients is not recommended as an alternative to strict infection-control procedures (OR=4.04; 95% CI 1.12-14.60) and correctly knew all HCV transmission patterns (OR=3.65; 95% CI 1.23-10.81) (Model 2 in Table 2).

**Table 3:** Attitudes about HCV infection prevention in HDU

**Attitudes**	**Agree%**	**Uncertain%**	**Disagree%**
HCWs are at risk of becoming infected with HCV by working in HDU (322)	**97.8**	0.6	1.6
Wearing gloves does not replace the need of hand washing (320)	**91.3**	1	8.7
Handwashing between patient contacts reduces the risk of HCV infection (320)	**81.6**	8.1	10.3
Transmission of bloodborne pathogens among HCWs may be prevented through adoption of evidence-based practices (309)	**80**	15.2	4.8
HCV can be spread from patient to patient in the HDU (312)	**70.8**	12.2	17

The number of subjects responding to the questions are in parentheses.

Bold indicates the correct answer.

*HCV* = hepatitis C virus.

*HDU* = hemodialysis units.

*HBV* = hepatitis B virus.

*HCWs* = healthcare workers.

Table 4 shows the respondents’ evidence-based practices for preventing HCV infection in HDU. Over 90% of the nurses **(**96.3% and 95%, respectively) reported wearing gloves when putting patients on and taking patients off of dialysis, but only half of the sample reported wearing gloves when touching care equipment and 29.2% of respondents did not wear gloves whenever providing patient care. Almost all nurses (98.8%) changed gloves for each patient, and 27.3% did not change gloves before administering intravenous medications; 62.5% reported always washing their hands after and before using gloves. 68.7% of respondents reported that medications were prepared in potentially contaminated areas.

**Table 4:** Respondents evidence-based practices for preventing HCV infection in HCWs and patients in HDU

	**Correct practice**	
	**N**	**%**
*Use of gloves*		
When putting patients on dialysis (322)	310	96.3
When taking patients off dialysis (322)	306	95
Whenever weighing patients (322)	285	88.5
Whenever providing patient care (322)	228	70.8
When preparing the machine (322)	205	63.7
When touching care equipment (322)	162	50.3
*Replacement of gloves*		
For each patient (322)	318	98.8
Before administering intravenous medications (322)	234	72.7
Whenever preparing the machine (322)	192	59.6
*Handwashing*		
Immediately after gloves are removed (320)	298	93.1
Immediately before gloves are weared (320)	214	66.9
Immediately after the machine is prepared (320)	212	66.2
*Handling of non disposable items and monitoring equipment*		
Nondisposable items and monitoring equipment cleaned and disinfected after use on one patient (299)	186	62.2
Dedicate to a single patient items that can not be easily disinfected (302)	97	32.1
*Individual protective equipment*		
Wear mask when splattering of blood onto face is possible (298)	255	85.6
Wear protective eyewear when splattering of blood into the eyes is possible (298)	243	81.5
Wear gown when splattering of blood onto clothes is possible (298)	174	58.4
Wear cap when splattering of blood onto head is possible (298)	91	30.5
Prepare medications in a room or area separated from the patient treatment area (310)	97	31.3

The number of subjects responding to the questions are in parentheses.

*HCV* = hepatitis C virus.

*HDU* = hemodialysis units.

After each HD session, 21% of respondents performed an improper disinfection procedure of visibly blood contaminated surfaces. When equipment management for HD was addressed, a high percentage of nurses reported that tourniquets and adhesive tapes were not dedicated to a single patient, and 44.6% and 24%, respectively, reused those items without cleaning and disinfecting (data not shown).

At bivariate analysis, appropriate handwashing procedures were significantly more likely in nurses with correct knowledge about isolation of HCV-infected patients (χ^2^=6.17, 1 df, p=0.013), and both at bivariate and multivariate analyses, in nurses who were aware that transmission of bloodborne pathogens among HCWs may be prevented through adoption of evidence-based practices (χ^2^=14.41, 1 df, p<0.001) (OR=3.02; 95% CI 1.60-5.69) and who reported a correct knowledge about HCV transmission patterns (χ^2^=12.02, 1 df, p=0.001) (OR=2.17; 95% CI 1.25-3.77) (Table 2 and Model 3 in Table 2).

## Discussion

This survey provides an outline of the state of knowledge, attitudes and compliance to evidence-based practices that prevent hospital transmission of HCV among nurses in a high-risk setting, the HDU, in one region of Italy.

In an era of clinical governance, it is important that HCWs are aware of the extent of HAI, and are thus able to take steps to minimize the complications of hospital care. Nurses, in particular, may be the first professionals to assess patients with HCV; therefore, raising the level of awareness on HCV infection among these health professionals may help to reduce the burden of disease.

### Nurses’ knowledge on evidence-based recommendations for preventing transmission of HCV in chronic HDU

As expected, the degree of knowledge about the most common transmission routes of HCV was satisfactory in our sample, whereas less than half (49.8%) of nurses correctly recognized all modes of HCV transmission. As previously stated, our hypothesis was that several practice characteristics, such as years in practice, specific education programs provided at HDU and satisfaction with equipment at HDU, might be determinants of knowledge and that several aspects of knowledge might be associated to each other. Our findings have partly confirmed the hypothesis, since a lower number of years in practice is associated with higher knowledge and two out of three variables investigating knowledge (knowledge about isolation of HCV-infected patients and knowledge that previous bloodstream infections should be included in medical record) were associated with our outcome variable on correct knowledge of all modes of transmission of HCV, whereas satisfaction with equipment at HDU and information about strategies to prevent HAI at the time of employment did not appear to be significant determinants of knowledge. This finding may be interpreted as a scarce effectiveness of information provided to HCWs in improving knowledge, or, since the mean years working in HDF was 11 years, information provided at the time of employment are no more relevant to discriminate knowledge of HCWs in our setting.

Lack of knowledge about the ineffectiveness of the use of dedicated dialysis machines and of the isolation of HCV-infected patients is worrying, since several studies reported that isolation of HCV-infected patients, as well as dialyzer reuse, did not influence HDU seroconversion rates [3,16,18-20]. A similar result has been observed in HCWs working in units using the central venous catheter, that were more knowledgeable about certain practices which are not recommended by any published guideline and whose purported efficacy is not strongly supported by current evidence [21]. This result appears even more surprising considering that in none of the surveyed HDU isolation of HCV patients and/or dedicated dialysis machines are available, and that the prevalence of HCV seropositive patients in Southern Italy is among the highest in Europe [22], and therefore, HCWs should be accustomed to appropriate procedures needed for their care. However, it seems that HCWs in dialysis facilities do not feel self-confident and comfortable in providing assistance to these patients, particularly for the opportunity to transmit the infection to other patients. This finding has already been reported by some of us for dentists and HIV seropositive patients [23] and nurses with HIV/HBV/HCV seropositive patients [24]. Moreover, they assumed the same procedures required for patients who test positive for hepatitis B surface antigen (isolation of patient with dedicated room and machines) should be applied for all viral hepatitis patients.

A high proportion of nurses knew that testing for HCV is essential when a patient first start HD to identify HCV transmission that occurs in HDU. Evidence-based guidelines recommend that all maintenance HD patients have to be screened for anti-HCV, since HCV infection is a significant cause of various forms of glomerulonephritis; in addition, testing for HCV offers the opportunity to provide antiviral treatment when appropriate. Several outbreaks have been identified due to screening and detection of seroconversions, and have prompted an evaluation of infection-control practices to correct lapses and prevent additional cases [10,25].

### Nurses’ attitudes toward evidence-based recommendations for preventing transmission of HCV in chronic HDU

In the present study, 97.8% of HCWs consider that they are at risk of becoming infected with HCV by working in HDU, whereas only 70% believe that HCV can be spread from patient to patient in HDU. This result shows an underestimation of the perceived risk from HCV in the patients, and an overestimation of the perceived risk in HCWs, posing the basis for paying more attention to protect themselves from bloodborne pathogens, rather than to patients. Similar suggestions come both from a survey among HCWs in ICU that perceived hand decontamination as a protection for themselves rather than for patients, performing it more frequently after patient contact [26]; and from a study in emergency departments that showed a lack of knowledge, particularly regarding infections that a HCW can transmit to a patient [27]. These findings are of concern since cross-infection from a HCV-infected patient to other simultaneously dialyzed patients is the principal mode of transmission of HCV in HDU [28].

The great majority of respondents agreed that transmission of bloodborne pathogens among HCWs may be prevented through adoption of evidence-based practices, and this result is in accordance with those of a cross-sectional census survey among nurses working in primary care that reported that almost 90% of respondents agreed that infection-control precautions would protect them from acquiring HCV [29].

Our hypothesis that solid knowledge and some practice characteristics might be determinants of positive attitudes has been partly confirmed. Indeed, findings clearly demonstrate that knowledge is a significant determinant of positive attitudes, since all variables exploring knowledge of HCWs (knowledge about HCV infection prevention, isolation of HCV-infected dialysis patients and HCV transmission patterns) are significantly associated to the positive attitude toward handwashing and wearing gloves, whereas practice characteristics do not seem to influence attitudes, since years in practice was not statistically significant and information about strategies to prevent HAI at the time of employment was excluded from the model. Thus, thorough knowledge of the recommended preventive strategies represents a first step in overcoming potential attitudinal barriers to compliance regarding recommendations for HCV infection prevention.

### Nurses’ evidence-based practices for preventing transmission of HCV in chronic HDU

We found that adherence to many aspects of recommended infection-control practices is adequate. However, some of the procedures under investigation deserve comments. Gloves are worn mainly for HCW self-protection, but permanent glove use can prejudice compliance with hand hygiene and may thus lead to cross-transmission of infectious agents. Therefore, it is essential to monitor that the recommendation that gloves always be worn in the HDU is followed properly, with systematic glove removal and hand hygiene between care procedures, to avoid exposure of patients to HCV transmission. The low adherence to gloves use when touching care equipment represents a serious gap, since there is evidence that the environment can serve as a reservoir for infectious virus [30,31]. This behavior is of concern since in HDU environmental surfaces and medical supplies are subjected to frequent blood contamination, therefore, it is vital for HCWs to wear all personal protective equipment. In our sample, although these two aspects appear to be adequately taken into account, it seems however that more attention is posed to procedures aimed at HCWs protection instead of those aimed at prevention of cross-contamination among patients.

An important observation elicited by the survey was that 62.5% of respondents wash their hands consistently with official KDIGO guidelines for infection-control precautions in HDU [11]. Hand hygiene represents the most basic and essential intervention for reducing HAI [32] and it is essential that everyone involved in care of maintenance HD patient is trained about infection prevention practices. Indeed, the risk of patient-to-patient infection substantially increases for lack of basic hand hygiene and failure to change gloves [33,34].

The interpretation of the results of the model aimed at exploring the role of predisposing factors, such as knowledge and attitudes, on correct handwashing practice as an indicator of overall correct preventive practice appears to be complex, since, although correct knowledge of HCV transmission patterns and awareness that transmission of bloodborne pathogens among HCWs may be prevented through adoption of evidence-based practices are significant predictors of correct handwashing practices, other two aspects of knowledge (knowledge about isolation of HCV-infected patients and about HCV prevention) and of positive attitudes (belief that wearing gloves does not replace the need of handwashing and awareness that handwashing between patient contacts reduces the risk of HCV infection) were not associated to this important preventive practice. It seems, to our opinion, that predisposing factors, although necessary, are not sufficient to predict practice, and more interest in enabling factors should be emphasized to improve compliance to correct preventive practice in this setting.

The important finding that only 31.3% of nurses work in a unit where medications are prepared in a room or area separated from the patient treatment area can be related to limited space rather than emphasis the management staff places on HCV infection control and prevention in the HDU.

### Limitations

There are some potential limitations in the design and measurements of this study that are worthy of emphasis and should be considered when interpreting the results. First, the analyses were based on cross-sectional data, and the findings should be interpreted with caution given the nature of the associations that limited us from drawing definitive causal conclusions about the observed relationships between independent characteristics and the outcomes measured. However, we feel that our study design was adequate to assess the knowledge, attitudes and evidence-based practices relating to transmission of HCV in HDU, in order to target tailored educational programs to nurses.

Second, self-reported responses, especially in relation to practices, can result in overestimation, thus introducing bias; and, without independent observation, it is unknown whether responses represent actual behavior. Direct observation was considered infeasible, as it was expensive, and it may also influence behavior. However, to gain a true reflection of knowledge and behavior, respondents were assured that their responses would have not been shared, since the questionnaires were anonymous.

Third, we collected data in one Italian region which might not represent all HCWs in Italy, and concern about generalizability and comparability of the findings may arise. However, we are confident that the findings of the study may be representative of the area surveyed and may be referred to the Southern part of our country.

Finally, it was not possible to identify characteristics of those who failed to return the questionnaire, so it was impossible to establish whether they were in any way different from those who did return it. However, there is no obvious reason to suspect that non-responders were substantially different from responders. Despite these limitations, the main advantage of the current study is the high response rate that excludes one major potential source of bias in the results. Response rate remains an important indicator of survey quality, and we believe that this high response rate was made possible by the extreme importance of the topic surveyed.

## Conclusions

In conclusion, the study findings support the existence of a potential causal chain: although changing behavior is a very complex process, correct knowledge on recommended infection-control practices appears to lead to changes in attitudes and contribute to improvement in practice. Behavior changes should be aimed at abandoning outdated practices and adopting and maintaining evidence-based practices. Initiatives focused at enabling and reinforcing adherence to effective prevention practices among nurses in HDU are strongly needed.

## Abbreviations

CI: Confidence interval; HAI: Healthcare-associated infections; HBV: Hepatitis B virus; HCV: Hepatitis C virus; HCWs: Healthcare workers; HD: Hemodialysis; HDU: Hemodialysis unit; ICU: Intensive care unit; OR: Odds ratio.

## Competing interests

The authors declare the absence of any interest to disclose and that the results presented in this paper have not been published previously in whole or part.

## Authors’ contributions

CP and CGAN participated in the design of the study, collected the data, and contributed to the data analysis and interpretation. AB and FB participated in the design of the study, collected the data, and contributed to the data analysis and interpretation and wrote the first draft of the article. MP, the principal investigator, designed the study, was responsible for the data analysis and interpretation, and wrote the article. All authors read and approved the final manuscript.

## Pre-publication history

The pre-publication history for this paper can be accessed here:

http://www.biomedcentral.com/1471-2334/13/76/prepub

## Supplementary Material

Additional file 1 Questionnaire. Click here for file

## References

[B1] WHO Hepatitis B Fact Sheet N°. 204 2008 Geneva: WHO http://www.who.int/mediacentre/factsheets/fs204/en/index.html

[B2] Sy T Jamal MM Epidemiology of hepatitis C virus (HCV) infection Int J Med Sci 2006 32 41 46 16614741 10.7150/ijms.3.41 PMC1415844

[B3] Petrosillo N Gilli P Serraino D Dentico P Mele A Ragni P Puro V Casalino C Ippolito G Prevalence of infected patients and understaffing have a role in hepatitis C virus transmission in dialysis Am J Kidney Dis 2001 37 1004 1010 10.1016/S0272-6386(05)80017-4 11325683

[B4] Saab S Brezina M Gitnick G Martin P Yee HF Jr Hepatitis C screening strategies in hemodialysis patients Am J Kidney Dis 2001 38 91 97 10.1053/ajkd.2001.25199 11431187

[B5] Fabrizi F Messa P Martin P Transmission of hepatitis C virus in hemodialysis: current concepts Int J Artif Organs 2008 31 1004 1016 19115192 10.1177/039139880803101204

[B6] Dyke M Hepatitis C in perioperative practice Br J Perioper Nurs 2002 12 18 23 11836981 10.1177/175045890201200102

[B7] Stein AD Makarawo TP Ahmad MF A survey of doctors’ and nurses’ knowledge, attitudes and compliance with infection control guidelines in Birmingham teaching hospitals J Hosp Infect 2003 54 68 73 10.1016/S0195-6701(03)00074-4 12767850

[B8] Roberts C Universal precautions: improving the knowledge of trained nurses Br J Nurs 2000 9 43 47 10887846 10.12968/bjon.2000.9.1.6412

[B9] Shimokura G Chai F Weber DJ Samsa GP Xia GL Nainan OV Tobler LH Busch MP Alter MJ Patient-care practices associated with an increased prevalence of hepatitis C virus infection among chronic hemodialysis patients Infect Control Hosp Epidemiol 2011 32 415 424 10.1086/659407 21515970 PMC3147181

[B10] Center for Disease Control and Prevention Recommendation for preventing transmission of infections among chronic hemodialysis patients MMWR Morbid Mortal Wkly Rep 2001 50 RR-5 1 46 11349873

[B11] KDIGO Clinical Practice Guidelines for the Prevention Diagnosis, Evaluation and Treatment of Hepatitis C in Chronic Kidney Disease Kidney Int 2008 73 Suppl 109 S1 99 10.1038/ki.2008.81 18382440

[B12] Center for Disease Control and Prevention Hepatitis C virus transmission at an outpatient hemodialysis unit-New York, 2001–2008 MMWR Morbid Mortal Wkly Rep 2009 58 189 194 19265779

[B13] Barnes S Concepcion D Felizardo G Moran J Peters V Shapiro S Yu M Guide to elimination of infections in hemodialysis: APIC elimination guide 2010 http://www.apic.org/Resource_/EliminationGuideForm/7966d850-0c5a-48ae-9090-a1da00bcf988/File/APIC-Hemodialysis.pdf

[B14] Yanai M Uehara Y Takahashi S Surveillance of infection control procedures in dialysis units in Japan: a preliminary study Ther Apher Dial 2006 10 78 86 10.1111/j.1744-9987.2006.00305.x 16556141

[B15] Shimokura G Weber DJ Miller WC Wurtzel H Alter MJ Factors associated with personal protection equipment use and hand hygiene among hemodialysis staff Am J Infect Control 2006 34 100 107 10.1016/j.ajic.2005.08.012 16630971

[B16] Jadoul M Cornu C Van Ypersele De Strihou C Universitaires Cliniques St-Luc collaborative Group Universal precautions prevent hepatitis C virus transmission: a 54 month follow-up of the Belgian multicenter study Kidney Int 1998 53 1022 1025 10.1111/j.1523-1755.1998.00823.x 9551413

[B17] Stata Corporation Stata Statistical Software: Release 11 2009 College Station TX: StataCorp LP

[B18] Finelli L Miller JT Tokars JT Alter MJ Arduino MJ National surveillance of dialysis-associated diseases in the United States, 2002 Semin Dial 2005 18 52 61 10.1111/j.1525-139X.2005.18108.x 15663766

[B19] Fissel RB Bragg-Gresham JL Woods JD Jadoul M Gillespie B Hedderwick SA Rayner HC Greenwood RN Akiba T Young EW Patterns of hepatitis C prevalence and seroconversion in hemodialysis units from three continents: the DOPPS Kidney Int 2004 65 2335 2342 10.1111/j.1523-1755.2004.00649.x 15149347

[B20] Dos Santos JP Loureiro A Cendoroglo Neto M Pereira BJ Impact of dialysis room and reuse strategies on the incidence of hepatitis C virus in hemodialysis unit Nephrol Dial Transplant 1996 11 2017 2022 10.1093/oxfordjournals.ndt.a027090 8918716

[B21] Bianco A Coscarelli P Nobile CGA Pileggi C Pavia M The reduction of risk in central line-associated bloodstream infections: knowledge, attitudes, and evidence-based practices in health care workers Am J Infect Control 2013 41 107 112 10.1016/j.ajic.2012.02.038 22980513

[B22] Touzet S Kraemer L Colin C Pradat P Lanoir D Bailly F Coppola RC Sauleda S Thursz MR Tillmann H Alberti A Braconier JH Esteban JI Hadziyannis SJ Manns MP Saracco G Thomas HC Trépo C Epidemiology of hepatitis C virus infection in seven European Union countries: a critical analysis of the literature. HENCORE Group (Hepatitis C European Network for Co-operative Research) Eur J Gastroenterol Hepatol 2000 12 667 678 10.1097/00042737-200012060-00017 10912488

[B23] Angelillo IF Villari P D’Errico MM Grasso GM Ricciardi G Pavia M Dentists and AIDS: a survey of knowledge, attitudes, and behavior in Italy J Public Health Dent 1994 54 145 152 10.1111/j.1752-7325.1994.tb01206.x 7932350

[B24] Angelillo IF Mazziotta A Nicotera G Nurses and hospital infection control: knowledge, attitudes and behaviour of Italian operating theatre staff J Hosp Infect 1999 42 105 112 10.1053/jhin.1998.0571 10389059

[B25] Centers for Disease Control and Prevention Hepatitis C virus transmission at an outpatient hemodialysis unit—New York, 2001–2008 MMWR Morbid Mortal Wkly Rep 2009 58 Suppl 8 189 194 19265779

[B26] Nobile CGA Montuori P Diaco E Villari P Healthcare personnel and hand decontamination in intensive care units: knowledge, attitudes and behavior in Italy J Hosp Infect 2002 51 226 232 10.1053/jhin.2002.1248 12144803

[B27] Parmeggiani C Abbate R Marinelli P Angelillo IF Healthcare workers and health care-associated infections: knowledge, attitudes, and behavior in emergency departments in Italy BMC Infect Dis 2010 10 35 43 10.1186/1471-2334-10-35 20178573 PMC2848042

[B28] Arenas MD Sanchez-Paya J Standard precautions in haemodialysis: the gap between theory and practice Nephrol Dial Transplant 1999 14 823 825 10.1093/ndt/14.4.823 10328450

[B29] Frazer K Glacken M Coughlan B Staines A Daly L Hepatitis C virus in primary care: survey of nurses’ attitudes to caring J Adv Nur 2011 67 Suppl 3 598 608 10.1111/j.1365-2648.2010.05516.x 21320157

[B30] Williams IT Perz JF Bell BP Viral hepatitis transmission in ambulatory health care settings Clin Infect Dis 2004 38 1592 1598 10.1086/420935 15156448

[B31] Hagan H Thiede H Weiss NS Hopkins SG Duchin JS Alexander ER Sharing of drug preparation equipment as a risk factor for hepatitis C Am J Public Health 2001 91 42 46 11189822 10.2105/ajph.91.1.42 PMC1446500

[B32] Steere AC Mallison GF Handwashing practices for the prevention of nosocomial infections Ann Intern Med 1975 83 683 690 1200507 10.7326/0003-4819-83-5-683

[B33] Savey A Simon F Izopet J Lepoutre A Fabry J Desenclos JC A large nosocomial outbreak of hepatitis C virus infections at a hemodialysis center Infect Control Hosp Epidemiol 2005 26 752 760 10.1086/502613 16209381

[B34] Delarocque-Astagneau E Baffoy N Thiers V Delarocque-Astagneau E Baffoy N Thiers V Simon N de Valk H Laperche S Couroucé AM Astagneau P Buisson C Desenclos JC Outbreak of hepatitis C virus infection in a hemodialysis unit: potential transmission by the hemodialysis machine? Infect Control Hosp Epidemiol 2002 23 328 334 10.1086/502060 12083237

