# Supplementary material for: Healthcare workers and prevention of hepatitis C virus transmission: exploring knowledge, attitudes and evidence-based practices in hemodialysis units in Italy
Source: BMC Infect Dis. 2013 Feb 7;13:76. doi: 10.1186/1471-2334-13-76 (PMC3571883; doi:10.1186/1471-2334-13-76)
Supplement: Additional file 1 — Questionnaire. [file 1471-2334-13-76-S1.doc]

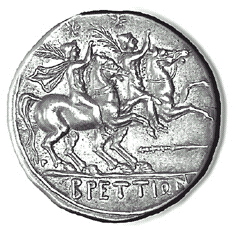
**DEPARTMENT OF HEALTH SCIENCES**

**MEDICAL SCHOOL**

**UNIVERSITY OF CATANZARO “MAGNA GRÆCIA”**

***HEALTHCARE WORKERS AND PREVENTION OF HEPATITIS C VIRUS TRANSMISSION: EXPLORING KNOWLEDGE, ATTITUDES AND EVIDENCE-BASED PRACTICES IN HEMODIALYSIS UNITS IN ITALY***

*Questionnaire used in the survey*

The purposes of the survey are to evaluate nurses’ knowledge, attitudes and frequencies of evidence-based practices that prevent hospital transmission of hepatitis C virus (HCV) in chronic hemodialysis units (HDU) in Calabria region (Italy).

According to Legislative Decree 2003/196 (Code for the protection of sensitive personal data), data will be analyzed only in an aggregate way and there will be no nominal data spreading. Precautions have been taken in order to guarantee confidentiality of gathered data and anonymity on respondents, so you can feel completely free to respond. Filling in and mailing back the questionnaire implicitly entails the approval for the use of data for the purposes of this survey.

**A. DEMOGRAPHICS AND PRACTICE CHARACTERISTICS**

| *a1*. YEAR OF BIRTH ________________ | *a2*. SeX M ❒ F ❒ |  |
| --- | --- | --- |
| ***A2*.** YEARs since BACCALAUREATE ___________ | | |
| ***A3.*** how long have you BEEN workING AS AN HEALTHCARE WORKER (HCW)? ________________ Months/Years | | |
| ***A4.*** how long have you BEEN workING in AN HDU? ________________ Months/Years | | |
| ***A5***. AT THE TIME OF EMPLOYMENT IN YOUR UNIT, WAS INFORMATION ABOUT STRATEGIES TO PREVENT HEALTHCARE ASSOCIATED INFECTIONS (HAI) PROVIDED TO YOU? 🞎 No 🞎 Yes | | |
| ***A6***. DURING PATIENT CARE DO YOU REFER TO SPECIFIC EVIDENCE-BASED HAI PREVENTION RECOMMENDATIONS? 🞎 No 🞎 Yes | | |
| ***A7***. ARE YOU SATISFIED ABOUT MEDICAL EQUIPMENT AVAILABLE IN THE HDU (CLAMPS, BLOOD PRESSURE CUFFS, TRAYS, TOURNIQUETS, ETC.)? 🞎 Completely dissatisfied 🞎 Fairly dissatisfied 🞎 Fairly satisfied 🞎 Completely satisfied 🞎 I do not know | | |

**B. CHARACTERISTICS OF HDU (TO BE COMPLETED ONLY BY HEAD NURSE)**

| ***B1*.** How many SHIFTS/DAY ARE PERFORMED IN THE HDU? ________________________ |
| --- |
| ***B2*.** How many PATIENTS/SHIFT ARE THERE IN THE HDU? ___________________ |
| ***B3.*** are CONTINUING EDUCATION PROGRAMS ABOUT STRATEGIES TO PREVENT HAI PROVIDED IN THE HDU? ❒ No ❒ Yes |
| ***B4*.** INFORMATION ABOUT STRATEGIES TO PREVENT HAI ARE PROVIDED TO: ❒ Patients ❒ Relatives ❒ Carers ❒ Nobody ❒ Others, Specify______________________ |
| ***B5.*** are the followING equipmentS adequateLY supplIED in the HDU?  ❒ Disposable gloves ❒ Biohazard containers ❒ Eyewears ❒ Disposable gown ❒ Masks ❒ Caps |
| ***B6.*** are BLOOD SAMPLES OR OTHER BODY FLUIDS COLLECTED IN DEDICATED AREAS? ❒ No ❒ Yes |
| ***B7.*** ARE PROTOCOLS ABOUT STRATEGIES TO PREVENT HAI AVAILABLE IN THE HDU? ❒ No ❒ Yes |

**C. KNOWLEDGE RELATED TO HCV INFECTION**

| ***C1.*** PLEASE INDICATE WHICH OF THE FOLLOWING ARE HCV TRANSMISSION PATTERNS**:** | | | | | | | | | | | |
| --- | --- | --- | --- | --- | --- | --- | --- | --- | --- | --- | --- |
| ❒ Hugging an HCV-positive individual ❒ Receiving a blood transfusion from an infected donor ❒ Ingestion of HCV-contaminated food ❒ Having sex with an HCV-positive partner ❒ Sharing needles while injecting drugs ❒ Kissing an HCV-positive individual ❒ Getting a tattoo ❒ Being born to a HCV-positive mother | | | | | | | | | | | |
| ***C2.*** PLEASE INDICATE WHICH OF THE FOLLOWING ARE HCV PREVENTION STRATEGIES: | | | | | | | | | | | |
| ❒ Avoid sharing razors or toothbrushes ❒ Avoid pregnancy ❒ Avoid kidney transplant ❒ Use a condom when having sex ❒ Avoid breastfeeding | | | | | | | | | | | |
| ***C3***. PLEASE INDICATE HOW MUCH YOU AGREE OR DISAGREE WITH THE FOLLOWING STATEMENTS | | | | | | | | | | | |
|  | | Strongly  Agree | | Agree | | Uncertain | | Disagree | | Strongly  Disagree | |
| ***C3.1*** Routine serologic testing for HBV and HCV infections should be included in medical record | **❒** | | **❒** | | **❒** | | **❒** | | **❒** | |  |
| ***C3.2*** Testing for HCV should be performed when patients first start hemodialysis | **❒** | | **❒** | | **❒** | | **❒** | | **❒** | |  |
| ***C3.3*** Testing for HCV should be performed periodically in hemodialysis patients | **❒** | | **❒** | | **❒** | | **❒** | | **❒** | |  |
| ***C3.4*** Vaccination against HBV should be included in medical record | **❒** | | **❒** | | **❒** | | **❒** | | **❒** | |  |
| ***C3.5*** Previous bloodstream infections should be included in medical record | **❒** | | **❒** | | **❒** | | **❒** | | **❒** | |  |
| ***C3.6*** Isolation of HCV-infected patients is recommended | **❒** | | **❒** | | **❒** | | **❒** | | **❒** | |  |
| ***C3.7*** The use of dedicated dialysis machines for HCV infected patients is recommended | **❒** | | **❒** | | **❒** | | **❒** | | **❒** | |  |

**D. ATTITUDES TOWARDS HCV INFECTION**

***D1***. PLEASE MARK FOR EACH OF THE FOLLOWING STATEMENTS THE ANSWER THAT BEST STANDS FOR YOUR VIEW

|  | | Strongly  Agree | | Agree | | Uncertain | | Disagree | | Strongly  Disagree |  |
| --- | --- | --- | --- | --- | --- | --- | --- | --- | --- | --- | --- |
| ***D1.1*** HCWs are at risk of becoming infected with HCV by working in HDU | **❒** | | **❒** | | **❒** | | **❒** | | **❒** | | |
| ***D1.2*** HCV can be spread from patient to patient in the HDU | **❒** | | **❒** | | **❒** | | **❒** | | **❒** | | |
| ***D1.3*** Transmission of bloodborne pathogens among HCWs may be prevented through adoption of evidence-based practices | **❒** | | **❒** | | **❒** | | **❒** | | **❒** | | |
| ***D1.4*** Wearing gloves replaces the need of hand washing | **❒** | | **❒** | | **❒** | | **❒** | | **❒** | | |
| ***D1.5*** Handwashing between patient contacts reduces the risk of HCV infection | **❒** | | **❒** | | **❒** | | **❒** | | **❒** | | |

**E. EVIDENCE-BASED PRACTICES FOR PREVENTING HCV INFECTION IN HDU**

| ***E1.*** IN WHICH OF THE FOLLOWING CIRCUMSTANCES DO YOU ALWAYS WEAR GLOVES? |
| --- |
| **❒** When putting patients on dialysis **❒** When taking patients off dialysis **❒** Whenever providing patient care **❒** When preparing the machine **❒** When touching care equipment  **❒** Whenever weighing patients |
| ***E2.*** IN WHICH OF THE FOLLOWING CIRCUMSTANCES DO YOU ALWAYS REPLACE GLOVES? |
| **❒** For each patient **❒** Before administering intravenous medications **❒** Whenever preparing the machine |
| ***E3.*** IN WHICH OF THE FOLLOWING CIRCUMSTANCES DO YOU ALWAYS WASH YOUR HANDS? |
| **❒** Immediately after gloves are removed **❒** Immediately before gloves are worn **❒** Immediately after the machine is prepared |
| ***E4.*** WHICH PROCEDURES DO YOU PERFORM FOR TREATMENT OF VISIBLY BLOOD CONTAMINATED SURFACES***? ________________________________________________________________________________________________________*** |
| ***E5.*** WHICH OF THE FOLLOWING PRACTICES DO YOU PERFORM DURING YOUR WORKING ACTIVITY? |
| **❒** I clean nondisposable items and monitoring equipment after use on one patient **❒** I disinfect nondisposable items and monitoring equipment after use on one patient **❒** I clean and disinfect nondisposable items and monitoring equipment after use on one patient **❒** I dedicate to a single patient items that can not be easily disinfected |
| ***E6.*** WHICH OF THE FOLLOWING INDIVIDUAL PROTECTIVE EQUIPMENT DO YOU WEAR WHEN SPLATTERING OF BLOOD IS POSSIBLE? |
| **❒** mask **❒** protective eyewear **❒** gown **❒** cap |
| *E7.* WHERE DO YOU PREPARE PATIENTS MEDICATIONS? |
| ❒ in the patient treatment area ❒ in a room separated from the patient treatment area❒ in an area separated from the patient treatment area |

**If you would like to add something more, please write it down in the space below**

______________________________________________________________________________________________________________________________________________________________________________________________________________________________________________________________________________________________________________________________

**Thank you for answering our questionnaire.**
